# Activity‐Informed Network Analysis Reveals Keystone Microbes Shaping Freshwater Ecosystem Function

**DOI:** 10.1111/1758-2229.70245

**Published:** 2026-03-19

**Authors:** Qiyao Yang, Rosa Aghdam, Patricia Q. Tran, Karthik Anantharaman, Claudia Solís‐Lemus

**Affiliations:** ^1^ Wisconsin Institute for Discovery, University of Wisconsin‐Madison Madison Wisconsin USA; ^2^ Department of Computer Science University of Wisconsin‐Madison Madison Wisconsin USA; ^3^ Department of Statistics University of Wisconsin‐Madison Madison Wisconsin USA; ^4^ Department of Bacteriology University of Wisconsin‐Madison Madison Wisconsin USA; ^5^ Department of Integrative Biology University of Wisconsin‐Madison Madison Wisconsin USA; ^6^ Department of Data Science and AI Indian Institute of Technology Madras Chennai India; ^7^ Department of Plant Pathology University of Wisconsin‐Madison Madison Wisconsin USA

**Keywords:** metagenomics/community genomics, microbe:microbe interactions, microbial ecology, microbiome

## Abstract

Freshwater lakes are dynamic ecosystems, with varying oxygen dynamics that influence microbiome structure, composition, and transcriptomic activity. In many freshwater studies, ecological function and abundance metrics are used to discover keystone species; however, it is well established that abundance does not equal activity. Despite the existence of long‐term time series spanning multiple years, no previous study has looked at how microbial community and activity (metatranscriptomics) are influenced by shifting oxygen conditions across depths at the microbial network level. In this study, we leverage metagenome‐assembled genomes and transcriptomic activity to identify keystone taxa in the ecosystem. Using the SPIEC‐EASI and CARlasso methods, we mapped key microbial associations and used permutation‐based analyses to assess the robustness of keystone identification. Our results reveal that a taxon's ecological centrality is context‐dependent and that many species identified as keystone by abundance alone do not exhibit corresponding transcriptional activity. Notably, members of Bacteroidota and other lineages emerged as keystone taxa only when both abundance and activity were considered. Our study underscores the importance of combining metagenomic and metatranscriptomic approaches for accurate identification of functionally relevant keystone species in freshwater ecosystems, providing a framework for future microbial ecology studies.

## Graphical abstract

Graphical abstract illustrating the workflow and key findings of this study. We first applied SPIEC‐EASI to metagenomic abundance data to construct microbial co‐occurrence networks and selected the top 15 MAGs based on degree centrality. We then used CARlasso to detect relationships between the top MAGs and environmental features. From this set, we selected one key MAG that exhibited strong environmental connectivity for further analysis. To assess the robustness of this key MAG, we performed a permutation‐based analysis by combining it with 14 randomly chosen MAGs and reconstructing networks across 100 permutations. The stability of the key MAG's role was quantified using the ‘connectivity robustness index’, which reflects how consistently it maintained high‐connectivity interactions across different community configurations. Notably, this key MAG showed high centrality in the metagenomic network but not in the metatranscriptomic network, highlighting that abundance‐based significance does not always align with transcriptional activity.

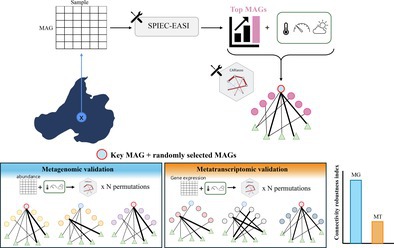

## 1 Introduction

Freshwater ecosystems are highly dynamic, shaped by fluctuating light regimes, temperature gradients, and oxygen availability, which collectively generate heterogeneous habitats for diverse microbial communities. These microorganisms are integral to ecosystem function, mediating critical processes such as nutrient cycling, biogeochemical transformations, energy transfer, and the maintenance of ecosystem stability (Falkowski et al. 2008b, 2008a; Arrigo 2005; Galloway et al. 2004; Worm and Duffy 2003). Advances in molecular biology and bioinformatics have enabled detailed characterisation of microbial community structure and function, elucidating how environmental variables including water temperature, dissolved oxygen, and dissolved organic matter modulate microbial diversity and activity (Handelsman 2004; Caporaso et al. 2012; Yvon‐Durocher et al. 2010; Liu et al. 2022; Tranvik 1998). A mechanistic understanding of these microbial dynamics is essential for predicting ecosystem responses to environmental change and for informing management and conservation strategies aimed at preserving the resilience and sustainability of freshwater environments. Recent progress in molecular and computational approaches has further illuminated the complex interplay of physical, chemical, and biological factors shaping microbial populations (Sunagawa et al. 2015; Shade et al. 2012; Bryanskaya et al. 2016; Li et al. 2024). Statistical models and network analyses have proven invaluable for uncovering co‐occurrence patterns and community responses to environmental perturbations, such as nutrient enrichment, and for identifying putative keystone taxa in freshwater lake microbiomes, including members of Microbacteriaceae, Rhodobacteraceae, and Sphingomonadaceae in the water column, as well as microalgae and protostomes in lake sediments, which are strongly linked to nutrient cycling and ecosystem stability (Ren et al. 2021; Li et al. 2023). With these insights, an important question arises: If network analyses were applied to activity‐based data rather than abundance data, would we identify the same keystone taxa? Addressing this question could fundamentally reshape our understanding of microbial community dynamics and their functional roles in aquatic ecosystems.

We applied this framework to reveal keystone species which might disproportionally influence the ecosystem in a model, dynamic lake ecosystem (Lake Mendota, WI, USA). Lake Mendota is a well‐studied seasonally anoxic, eutrophic freshwater lake located in the North American Midwest (Zhou et al. 2025; Rohwer et al. 2025). It is part of the North‐Temperate Lakes Long‐Term Ecological Research (NTL‐LTER) (https://lter.limnology.wisc.edu/) and maintains a record of multiple decades of limnological data. In terms of microbial ecology, it is home to the Microbial Observatory, which collects weekly integrated water column samples (mixing the upper 12 m of the water column) during the open‐water period (Rohwer and McMahon 2022). While routine water column sampling has provided valuable insights into community composition over time, it has offered limited resolution on depth‐specific activity. In 2020, we set up a parallel sampling program to collect paired virome‐metagenome‐metatranscriptomes samples (Tran et al. 2023). Over 100 depth‐discrete paired DNA and RNA samples were collected, and 30 samples representative of oxygen shifts were selected for sequencing, and are fully described in (Tran et al. 2023). Building upon this dataset, in this study, we seek to address the question of keystone species and network stability in the context of their abundance and activity in the microbial community (see Tran et al. 2023 for detailed methodologies).

In this study, we seek to fundamentally redefine how keystone microbial species are identified and validated within dynamic aquatic ecosystems. By leveraging advanced computational tools such as Sparse Inverse Covariance Estimation for Ecological Association Inference (SPIEC‐EASI) (Kurtz et al. 2015) and the Chain Graph Least Absolute Shrinkage and Selection Operator (CARlasso) (Shen and Solis‐Lemus 2020, 2021), we construct high‐resolution Gaussian graphical models to map microbial community networks and reveal how these networks are reshaped by environmental changes, including shifts in salinity, temperature, and nutrient availability. To rigorously test the stability of identified keystone species, we employ iterative network simulations, running hundreds of permutations for each metagenome‐assembled genome (MAG) to distinguish between context‐dependent taxa and those that consistently maintain their central roles. Crucially, we bridge the gap between microbial abundance and functional activity by integrating paired metatranscriptomic data, allowing us to validate whether taxa identified as keystone in abundance‐based networks are also transcriptionally active and functionally important. This multi‐layered approach challenges traditional paradigms in microbial ecology and examines whether keystone species are artefacts of detection methods or true drivers of ecosystem function, potentially reshaping our understanding of microbial community dynamics and their ecological significance.

## 2 Results

### 2.1 Environmental Conditions Represented in the Lake Mendota Samples

**FIGURE 1:**
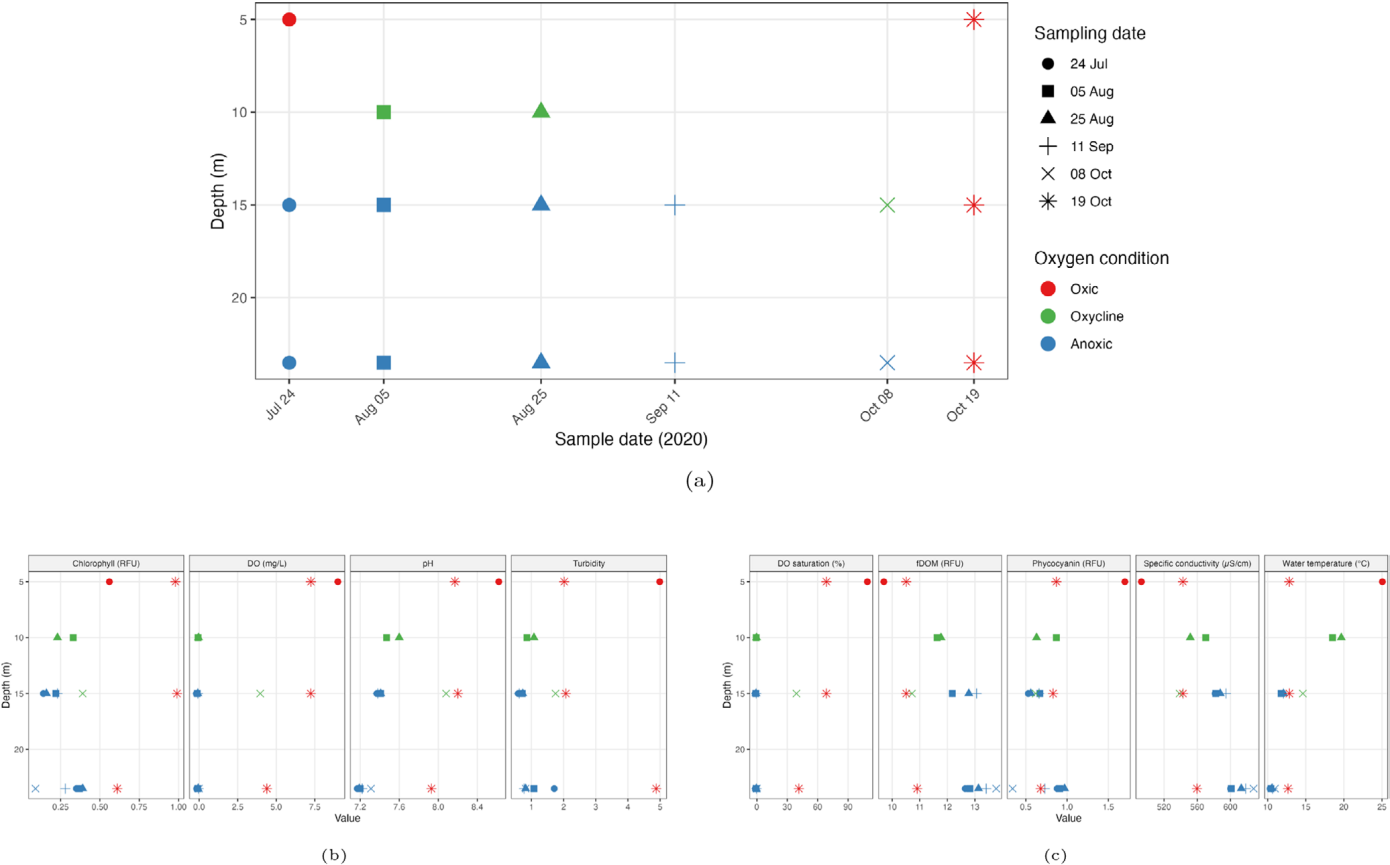
Environmental conditions of Lake Mendota in 2020. (a) Sampling grid across dates and depths by oxygen regime. (b, c) Values of nine environmental features. Across all panels, different colours represent oxygen levels, and different shapes denote sampling dates. For additional details on the data, see figure 1 in Tran et al. (2023).

Lake Mendota was sampled during summer and early fall 2020 at multiple depths. We measured 11 environmental variables: depth, water temperature (°C), dissolved oxygen saturation (%), dissolved oxygen concentration (mg/L), specific conductivity (μS/cm), pH, chlorophyll (RFU), phycocyanin (RFU), fDOM (RFU), turbidity (FNU), and sampling time. Figure 1 shows the environmental setting of the study and these measurements provide the background context for the microbial community analyses. We analysed 16 samples collected from Lake Mendota during the summer and early fall of 2020 to investigate microbial dynamics across different environmental conditions. Samples were sequenced at four depths (5 m [*n* = 2], 10 m [*n* = 2], 15 m [*n* = 6], and 23.5 m [n = 6]) and spanned 4 months (July [*n* = 3], August [*n* = 6], September [*n* = 2], October [*n* = 5]). Samples were classified based on the oxygen conditions at the time of sampling (Oxic [*n* = 4], Anoxic [*n* = 9], Oxycline [*n* = 3]). Together, these groupings represented 11 distinct environmental conditions. Figures S1 and S2 show MAG abundance distributions before and after total sum scaling (TSS) normalisation, and Figures S3 and S4 present relative abundances by environmental conditions. Sample labels follow the format ‘Month–Day–Depth–Oxygen’ to ensure consistency across figures. To evaluate microbial co‐occurrence patterns among the 16 samples, we performed correlation analyses using both Pearson's (for linear relationships) and Spearman's (rank‐based, for monotonic relationships) methods on the relative abundance profiles of our 431‐MAG library, which yielded consistent correlation values across the samples, thereby confirming robust associations regardless of the method used. This consistency underscores the reliability of our findings and is visually summarised in Figure S5 for Pearson correlation and Figure S6 for Spearman correlation. We focused on strong positive correlations (*r* > 0.8) that were shared across both metrics. Several sample pairs showed high agreement between the two methods, particularly among samples from August and September, as well as among October samples across different depths. A full list of intersecting sample pairs with strong correlations is provided in Table S1. Anoxic samples from August 25 and September 11 at both 15 and 23.5 m showed consistently high correlations, reflecting their close temporal and depth similarity. The 15 and 23.5 m anoxic samples from September 11 were also strongly correlated. In October, correlations spanned depths: the October 8 sample at 15 m (oxycline) was highly similar to October 19 oxic samples from 5, 15, and 23.5 m, which were themselves strongly correlated.

### 2.2 Overview of MAG Diversity and Assemblages

**FIGURE 2:**
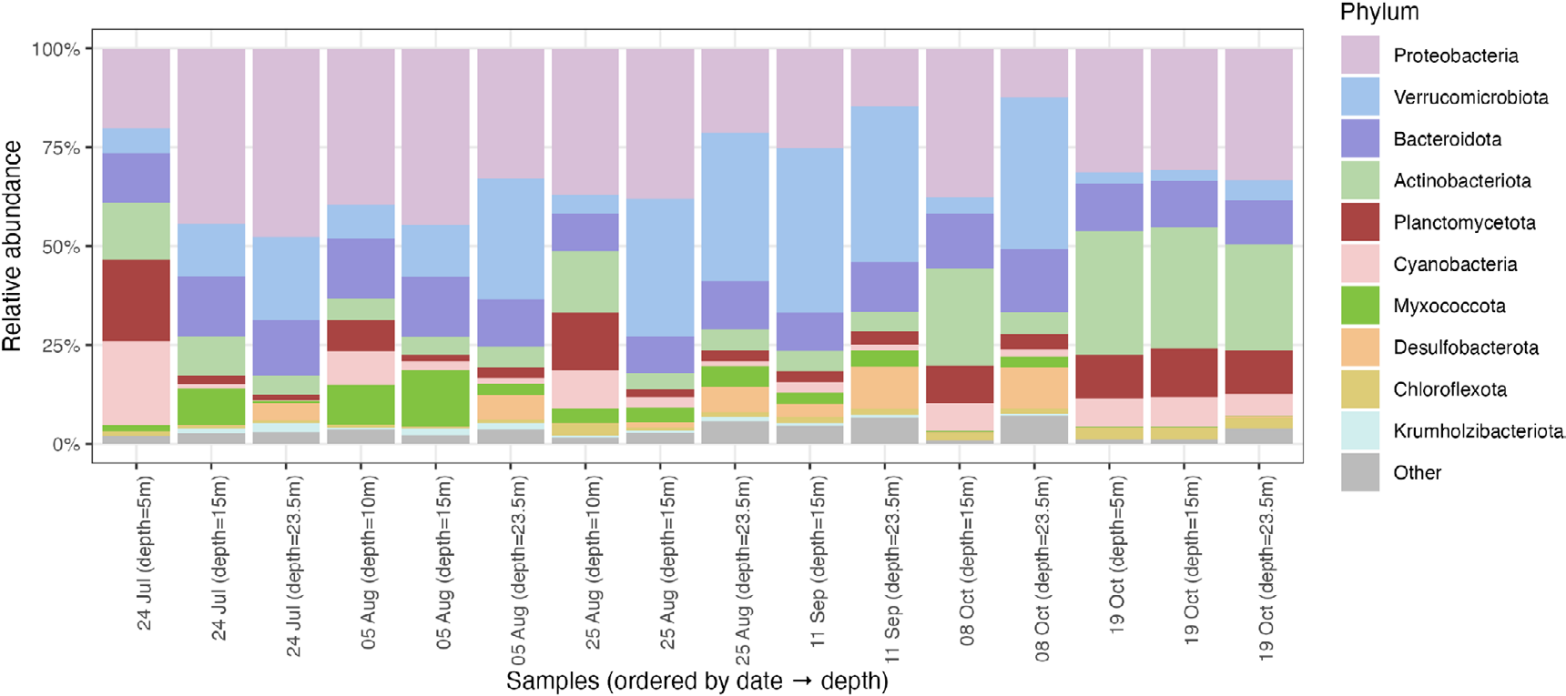
Phylum‐level composition of MAGs assemblages across Lake Mendota samples. Bars show relative abundance after total‐sum scaling of read counts. Samples are ordered by sampling date and then depth and labelled ‘DD Mon (depth=X m)’. Only the 10 most abundant phyla across the dataset are shown explicitly; all remaining lineages are grouped as *Other*.

Our analysis centers on 431 MAGs, employing various statistical tools to explore the relationships between microbial taxa. These MAGs span a wide taxonomic range. At higher taxonomic levels, nearly all MAGs could be classified, with assignments across 27 phyla, 54 classes, and 104 orders (Table 1). Proteobacteria were the most common phylum (100 MAGs), with Gammaproteobacteria (80 MAGs) representing the dominant class. Within the orders, Burkholderiales was the most represented (61 MAGs). At finer levels, taxonomic resolution decreased: only 417 MAGs could be assigned at the family level, 349 at the genus level, and just 44 at the species level. The most frequent family was Burkholderiaceae (31 MAGs), while the most common genus was Planktophila (11 MAGs). This decline in classification depth highlights both the novelty of the recovered MAGs and the limitations of current reference databases, especially at lower ranks. Together, these results underscore the broad phylogenetic diversity of the dataset and provide important context for subsequent analyses of community composition and network centrality. To contextualise diversity across samples, we summarised MAG composition at the phylum level for each sequenced sample (Figure 2). Bars are total‐sum scaled (relative abundance), samples are ordered by date then depth and labelled ‘day month (depth=Xm)’, and only the top phyla are shown (others grouped as ‘Other’). Proteobacteria are consistently dominant, while Verrucomicrobiota, Bacteroidota, and Actinobacteriota vary across dates and depths; lower‐abundance lineages (e.g., Planctomycetota, Cyanobacteria, Myxococcota, Chloroflexota, Desulfobacterota) contribute modestly to the ‘Other’ fraction.

**TABLE 1:** Summary of taxonomy for the 431 MAGs.

Taxonomic resolution	Unique known	Known MAGs	Unknown MAGs	Most frequent known	Count
Domain	2	431	0	Bacteria	429
Phylum	27	431	0	Proteobacteria	100
Class	54	431	0	Gammaproteobacteria	80
Order	104	430	1	Burkholderiales	61
Family	151	417	14	Burkholderiaceae	31
Genus	176	349	82	Planktophila	11
Species	43	44	387	Sediminibacterium sp002299885	2

*Note:* The table shows the number of unique taxa identified at each level, the number of MAGs with known versus unknown assignments, and the most frequent classified lineage with its count. While nearly all MAGs could be classified at higher levels (domain, phylum, class), resolution decreased toward finer levels, with only 44 MAGs assigned at the species level.

### 2.3 Microbial Community Variation Across Enviromental Conditions

**FIGURE 3:**
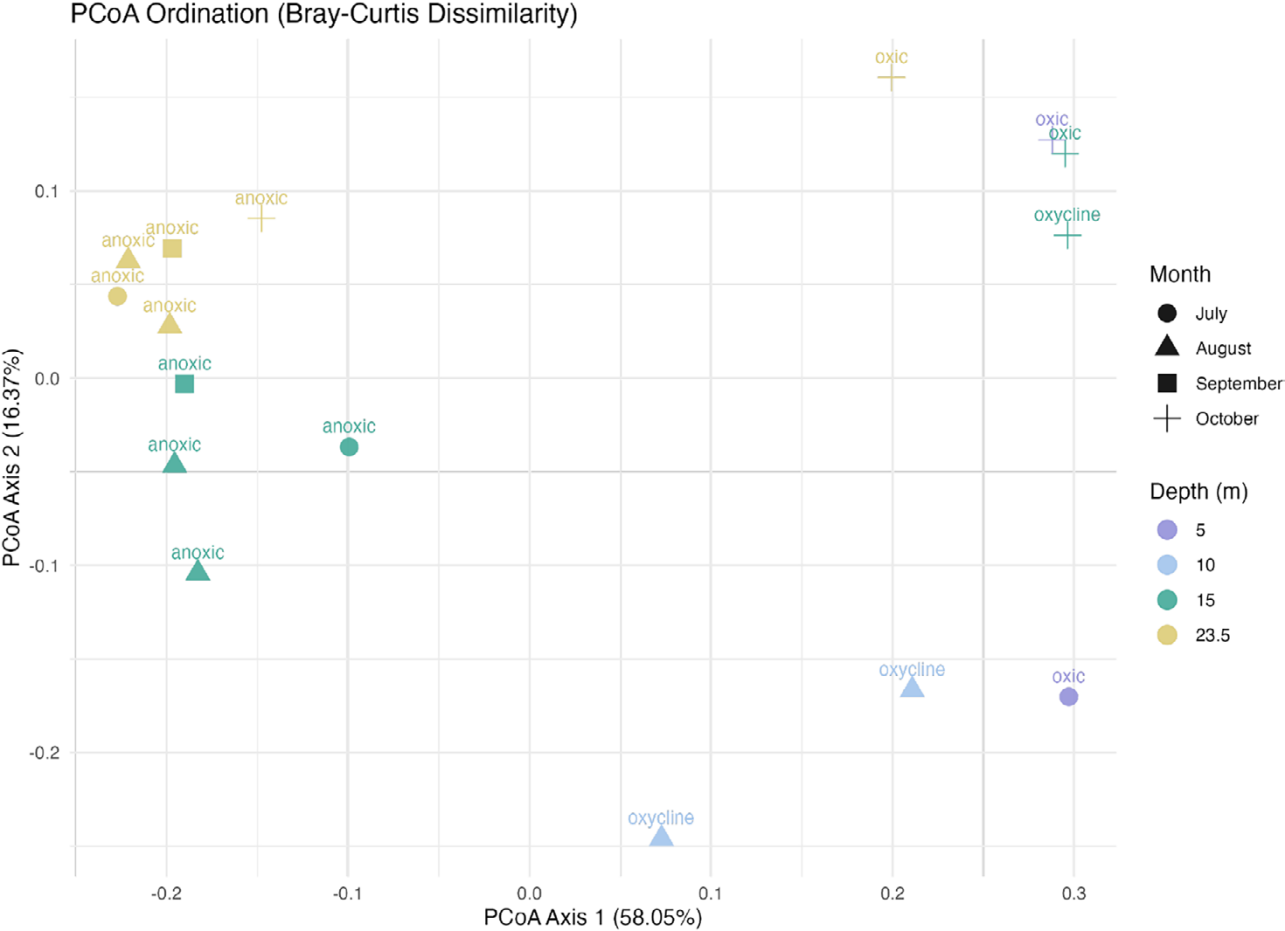
Principal coordinates analysis (PCoA) ordination of microbial communities based on Bray–Curtis dissimilarity. The Bray–Curtis distance matrix was calculated from Hellinger‐transformed abundance data to reduce the influence of dominant taxa and enhance comparability across samples. The first two axes (PCoA1 and PCoA2) explain 58.05% and 16.37% of the total variance, respectively. Points are coloured by sampling depth and shaped by sampling month, with each sample labelled according to its oxygen level.

To investigate spatial and temporal patterns in microbial community composition, we performed a principal coordinates analysis (PCoA) and permutational multivariate analysis of variance (PERMANOVA). The PCoA plot (Figure 3) illustrates gradients in microbial community composition across samples, with the first two principal coordinates (PCoA1 = 58.05%, PCoA2 = 16.3%) jointly explaining 74.42% of the variance. Samples located near one another in ordination space exhibited more similar microbial compositions. PERMANOVA based on Bray–Curtis dissimilarity revealed significant differences in community composition across depth (*p* = 0.018, *R* ^2^ = 0.39), month (*p* = 0.028, *R* ^2^ = 0.38), and oxygen conditions (*p* = 0.001, *R* ^2^ = 0.60). A combined model incorporating all three factors (depth, month, and oxygen) was significant (*R* ^2^ = 86%, *p* = 0.001). Interaction models also revealed significant effects, including depth × month (*p* = 0.005), month × oxygen (*p* = 0.001), and depth × oxygen (*p* = 0.002). The three‐way interaction depth × month × oxygen was also significant (*p* = 0.016). Pairwise PERMANOVA comparisons further confirmed strong differences between 5 and 23.5 m (*p* = 0.034) and between 23.5 and 10 m (*p* = 0.043). For temporal variation, October samples differed significantly from August (*p* = 0.013), and marginally from September (*p* = 0.054), while other seasonal comparisons were not significant (*p* > 0.1). Among oxygen conditions, microbial communities significantly differed between oxic and anoxic (*p* = 0.003) and between anoxic and oxycline (*p* = 0.002).

### 2.4 Most Abundant MAGs Across Samples

**FIGURE 4:**
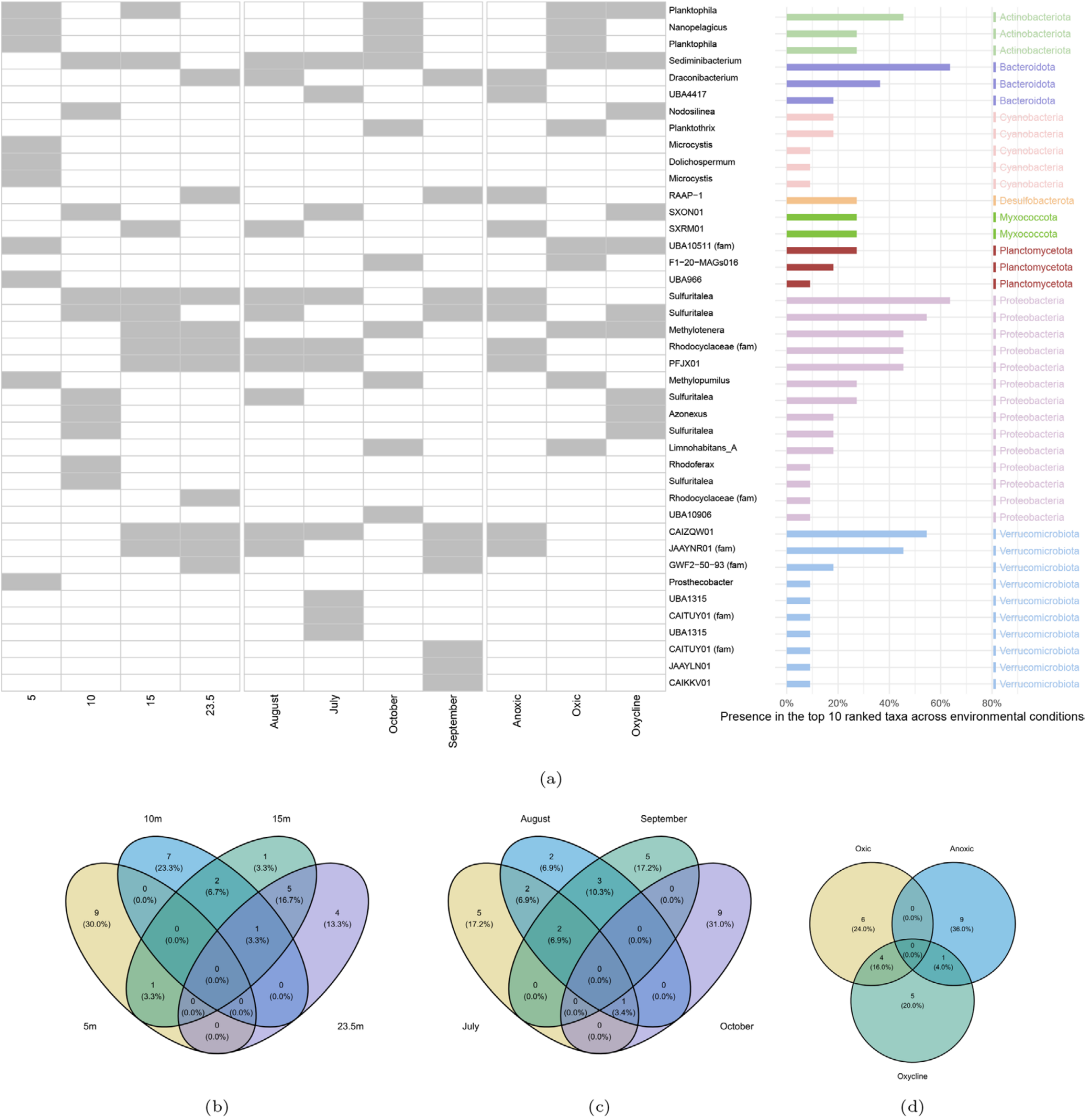
Overview of the most frequently abundant MAGs across depths, months and oxygen levels. (a) Presence of top 10 MAGs across 11 environmental conditions, ordered by Phylum. The heatmap displays the binary presence (grey) or absence (white) of the top MAGs identified across 11 environmental conditions, including four depths (5, 10, 15, 23.5 m), 4 months (July–October), and three oxygen levels (oxic, anoxic, oxycline). Rows are labelled by the Genus name when available; unclassified MAGs are labelled at the lowest confident taxonomic level (e.g., Family (fam) or ‘Unclassified’). MAGs are grouped by Phylum, with each Phylum colour‐coded in the accompanying bar chart. The accompanying horizontal bar chart indicates the percentage of conditions (out of 11) in which each MAG was present. MAGs are sorted within each Phylum by decreasing prevalence. (b) Venn diagram showing the overlap of the top 10 MAGs across four depth layers (5, 10, 15, and 23.5 m). (c) Venn diagram showing the overlap of the top 10 MAGs across four monthly sampling points (July, August, September, and October). Two MAGs, ‘Ga0485157_metabat1.059 (g_Sulfuritalea)’ and ‘Ga0485167_maxbin.109 (g_CAIZQW01)’ are important in 3 months. (d) Venn diagram showing the overlap of the top 10 MAGs across three oxygen levels (oxic, anoxic, and oxycline).

Having established the overall community composition, we next quantified how frequently each of the most abundant MAGs appeared across environmental conditions, using the procedure described in Section 4.1.3. The results of this analysis are provided in a Supplementary table on GitHub (https://github.com/solislemuslab/lake‐microbiome‐data‐analysis/), which lists each MAG's taxonomy (Phylum–Class–Order–Family–Genus; genus reported when available), its presence across the 11 environmental categories, and two summary metrics: Presence_Count (number of categories, out of 11, in which the MAG appears among the top 10) and Bar_Percent (Presence_Count/11 × 100; the value shown in the bar chart in Figure 4a). In total, 41 MAGs belonging to eight distinct phyla were identified as top‐ranked across conditions (Figure 4a). The less frequent top 10 MAGs were found in only one of the 11 environmental conditions, whereas consistently highly ranked MAGs occurred in up to seven conditions. Notably, Verrucomicrobiota were among the most frequently top taxa, but were limited to the anoxic hypolimnion of the lake. Previous studies, however, have reported Verrucomicrobiota as important in surface waters of Lake Mendota (Rohwer et al. 2025; Marick et al. 2021), while other studies have shown that they can also be abundant in deep hypolimnia (Lindström et al. 2004). Since the long‐term time series encompasses the hypolimnetic waters during the stratified portions of the year, those samples could have included Verrucomicrobia mixed from the deep waters, resulting in that finding. To better understand how environmental conditions structure microbial communities, we also examined overlaps among top‐ranked MAGs across depths (Figure 4b), months (Figure 4c), and oxygen levels (Figure 4d) using Venn diagrams.

### 2.5 Using Bayesian Sparse Networks to Identify Connections Between MAGs and Environmental Conditions

#### 2.5.1 Identifying Central MAGs in Microbial Community Networks

**FIGURE 5:**
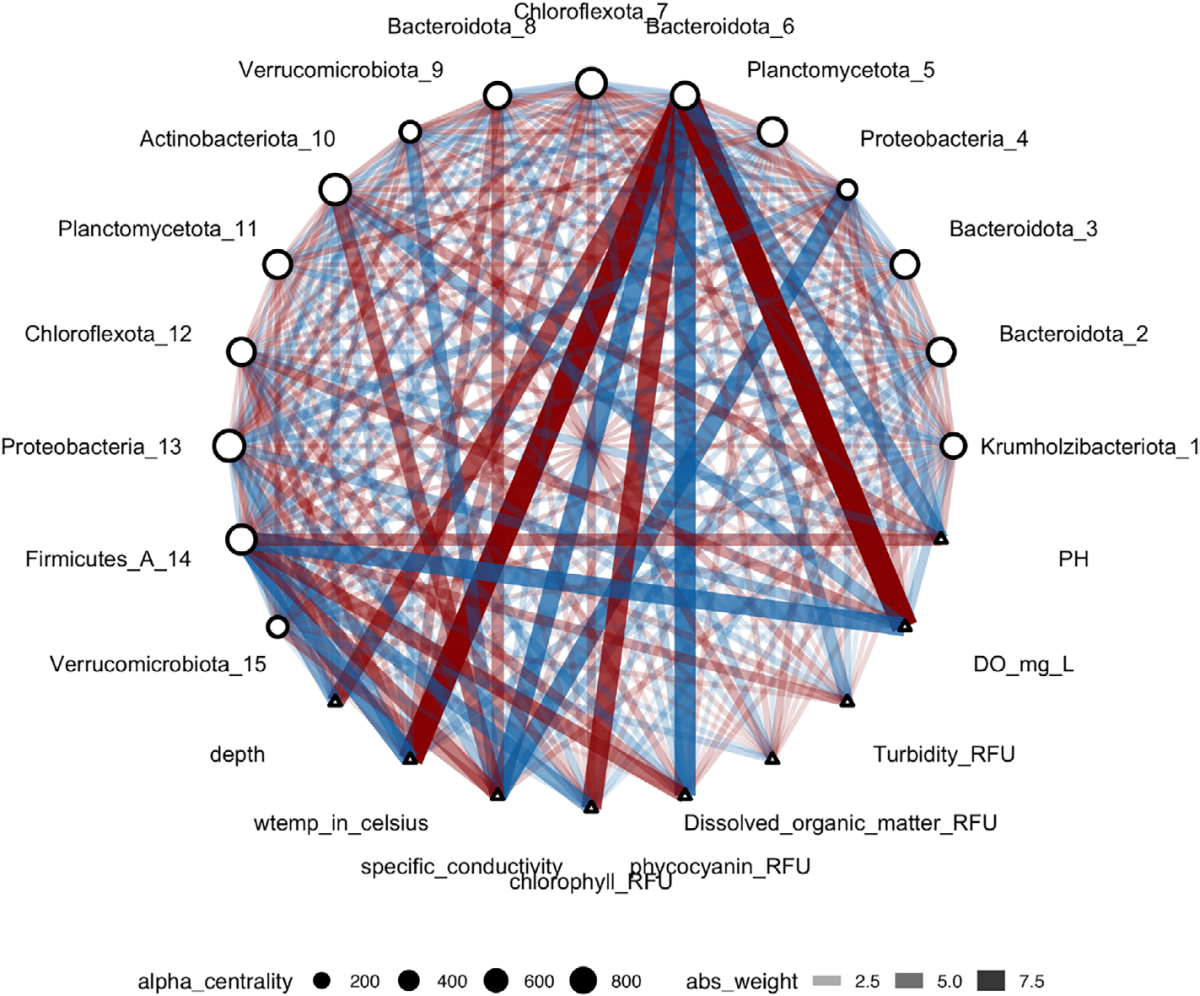
CARlasso network diagram showing the top 15 nodes with the highest degree centrality, selected using the SPIEC‐EASI algorithm (Kurtz et al. 2015), and their connections to environmental conditions. The nodes, representing MAGs categorised by Phylum, and environmental conditions, are connected based on the CARlasso analysis (Shen and Solis‐Lemus 2020, 2021). Node size indicates degree centrality and edge thickness reflects interaction strength. The edge colour indicates the type of relationship, with blue representing negative relations and red representing positive relations. Environmental conditions include physical and chemical variables such as depth, water temperature (wtemp), dissolved oxygen (do_raw), specific conductivity (sp_cond), pH, and fluorescence‐based proxies including chlorophyll (chlor_rfu), phycocyanin (phyco_rfu), dissolved organic matter (fdom_rfu), and turbidity (turb_fnu) (see Table S3).

**FIGURE 6:**
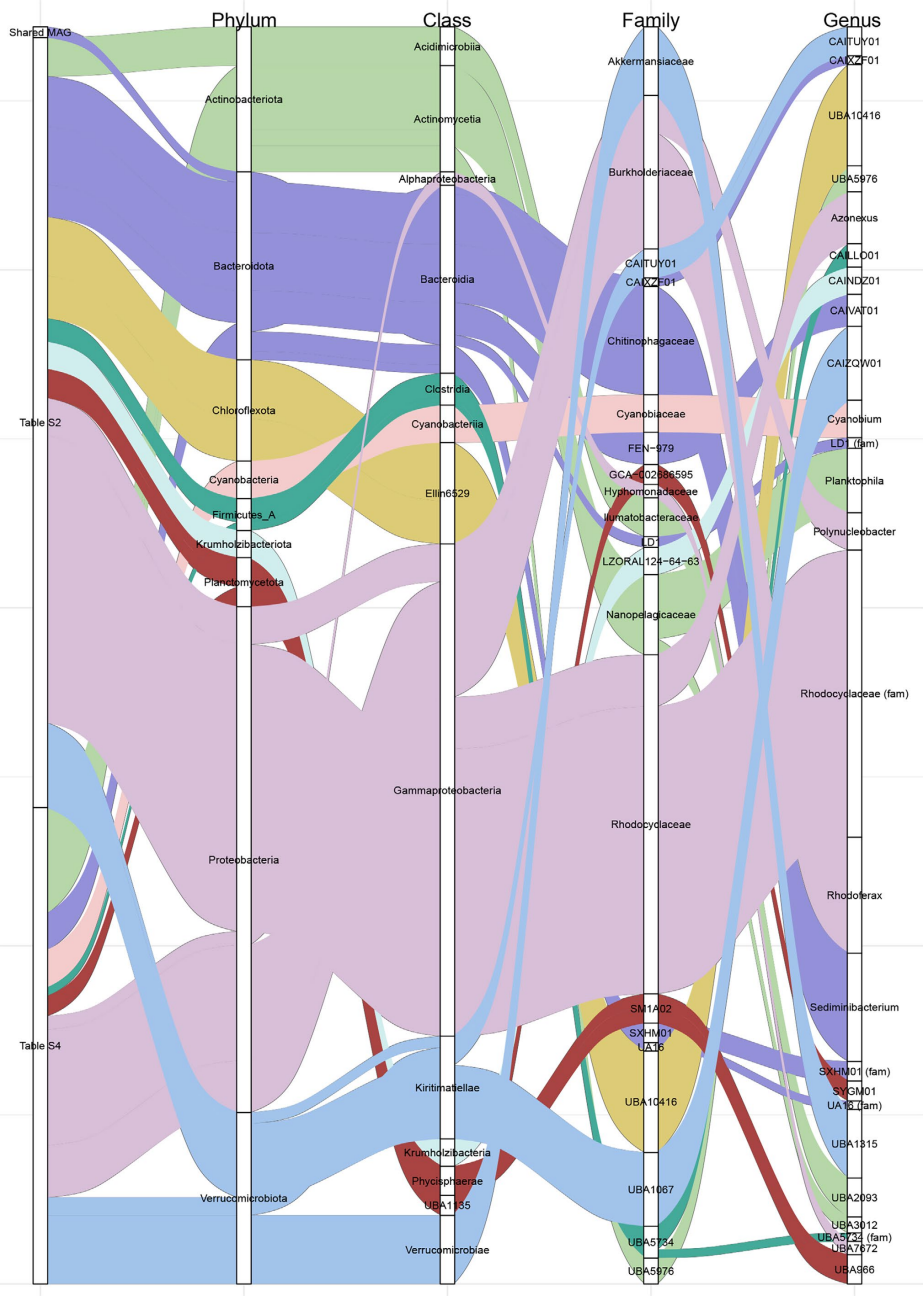
Multi‐level taxonomic distribution of 29 MAGs from Tables S2 and S4. Multi‐level taxonomic distribution of 29 MAGs from Table S2 (top 15 nodes with the highest degree centrality, from the CARlasso network, Figure 5) and Table S4 (MAGs from the permutation analysis, Figure 7). The Sankey diagram traces each MAG from group assignment to its taxonomy (phylum, class, family, genus). Edge widths represent the average relative abundance of each MAG across samples. If a MAG belongs to multiple categories at the next taxonomic level (e.g., a Phylum connecting to multiple classes), the edge width is proportionally divided. One MAG (Bacteroidota_6 or Ga0485171_metabat1.063) is shared between both tables and appears under the ‘Shared MAG’ group.

We performed SPIEC‐EASI, as detailed in Section 4.2 to identify the MAGs with the highest degree of centrality (most connections). We then constructed a Bayesian sparse microbial network via CARlasso (Figure 5). In this figure, the most connected nodes represent putative engagement with other microbes and environmental conditions. Of note, Bacteroidota_6 (Ga0485171_metabat1.063) was the top‐ranked node among the 15 with the highest degree centrality (Table S2) and was selected for downstream analyses (Figure 6).

#### 2.5.2 How Does Microbial Community Change Impact the ‘Centrality’ of a MAG

**FIGURE 7:**
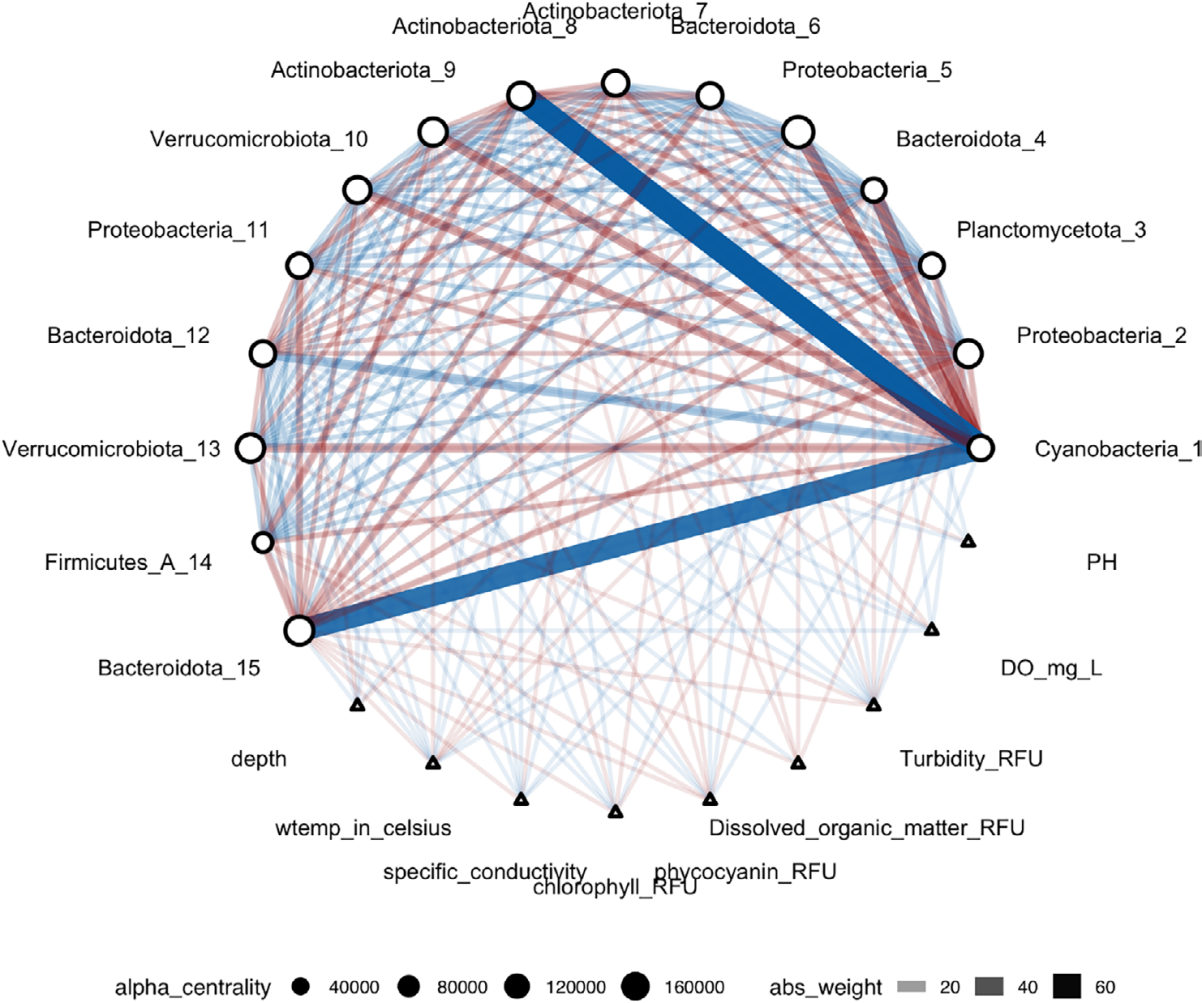
Evaluating the network role of Bacteroidota_6 under community variation. This network diagram shows the results of an analysis in which Bacteroidota_6 (a highly central MAG identified in Figure 5) was retained, while 14 other MAGs were randomly selected to represent alternative community compositions. Node size indicates degree centrality, while edge thickness reflects interaction strength; red edges denote positive associations and blue edges indicate negative associations. Nodes represent MAGs and environmental conditions, including depth, water temperature (wtemp), dissolved oxygen (do_raw), specific conductivity (sp_cond), pH, and fluorescence‐based proxies including chlorophyll (chlor_rfu), phycocyanin (phyco_rfu), dissolved organic matter (fdom_rfu), and turbidity (turb_fnu) (see Table S3).

In the natural environment, community structure and composition are expected to change as a result of environmental change. To evaluate the robustness of a MAG's centrality, we simulated changes in community structure by fixing a highly central MAG and randomly selecting 14 other MAGs to construct alternative networks. For example, take MAG Bacteroidota_6 (Ga0485171_metabat1.063), which was identified as a central MAG. We observed a significant change with its previously strong connections to environmental conditions such as dissolved oxygen (do_raw) and water temperature (wtemp) dissipating in these analyses (Figure 7). A comprehensive list of the MAG identities used in this permutation analysis is provided in Table S4.

The taxonomic identities of the 29 key microbes in our analysis were resolved to understand the composition of the core community. These MAGs comprising the top 15 most central nodes, whose genomic details are listed in Table S2, and the MAGs from the permutation analysis identified in Table S4 were taxonomically diverse, spanning 11 different phyla. This distribution from Phylum to Genus is visualised in a Sankey diagram (Figure 6). The width of each connection reflects the average relative abundance of the corresponding MAG. If a MAG was linked to multiple downstream taxonomic categories (e.g., a single Phylum connecting to multiple classes), edge widths were proportionally divided. Only one MAG (Bacteroidota_6) appeared in both groups and is shown under the ‘Shared MAG’ category.

#### 2.5.3 Validating Keystone Status Through Activity

The connectivity robustness index described in Section 4.3 for Bacteroidota_6 in the metagenomic network was satisfied in 42%, while in the metatranscriptomic network, it was met in 44% of analyses. These findings suggest that Bacteroidota_6 is not only structurally embedded in microbial communities (as shown by its genomic presence) but also functionally active in gene expression (as shown by its transcriptomic significance), reinforcing its role as a potential keystone taxon. A similar analysis was performed for Verrucomicrobiota_9 (Ga0485167_metabat2_ours.023), which exhibited a considerably lower connectivity robustness index: 11% in metagenomic and 6% in metatranscriptomic permutations.

## 3 Discussion

### 3.1 Microbial Community Composition Influences the Identity of Keystone Taxa

We identified 15 microbial taxa with the highest connectivity using SPIEC‐EASI and examined their interactions with environmental conditions through the CARlasso network (Figure 5), highlighting their central role in microbial community dynamics. These findings underscore that the importance of key taxa is not universal but depends on the surrounding microbial community. To test this, we reassembled communities by retaining a keystone MAG and randomly selecting other members. Two patterns emerged: the keystone microbe either maintained its centrality or lost it, with other taxa sometimes emerging as important. This context dependence was exemplified by Bacteroidota_6 (Ga0485171_metabat1.063). Although initially highly central, its strong connections to environmental conditions dissipated in alternative community settings (Figure 7). As its influence declined, other MAGs rose in prominence, illustrating how microbial network structure can shift with compositional changes. This is further demonstrated in Figure 7, where the reassembly analysis emphasises how biological interactions can sometimes outweigh environmental conditions in shaping microbial network dynamics. This stability, referring to the ability of microbial communities to maintain key taxa across different environmental conditions, highlights the complexity of microbial ecosystems and the dynamic interplay between community structure and environmental change. Recent work, including a comprehensive review by Tudela et al. (2021), has further explored the indispensable roles of keystone species within the gut microbiota, key regulators of metabolic functions and host health. These keystone species significantly influence the stability of microbiomes; their presence can drastically alter microbiome architecture and, consequently, affect the host's health profoundly. Adding to this, Xun et al. (2021) focused on soil microbiomes, demonstrating how keystone taxa with specialised metabolic functions, such as nitrogen and phosphorus metabolism, enhance community stability. The findings reveal that these keystone taxa play a pivotal role in maintaining soil health and fertility. Moreover, recent research highlights the essential role of keystone taxa in the recovery of the human gut microbiome after antibiotic treatment, identifying specific bacteria that facilitate rapid ecological recovery and resilience (Gibbons 2020). This underscores the broader ecological principle that keystone taxa are not only crucial for maintaining balance and health in natural ecosystems but also play a fundamental role in human health, particularly in the resilience and stability of our internal microbial communities. In future work, we plan to replace the SPIEC‐EASI results with a consensus network framework and to strengthen keystone taxa identification by incorporating more reliable taxa selection methods (Aghdam et al. 2025; Aghdam and Solís‐Lemus 2025).

### 3.2 Metabolic Basis for Interactions

Microbial interactions within complex communities are fundamentally driven by metabolic exchanges, where the transformation and release of compounds by one organism create opportunities for others to thrive. In the anoxic zone of Lake Mendota, our network analysis identified two highly connected keystone species from distinct phyla: Bacteroidota_6 and Verrucomicrobiota_9. Both exhibit overlapping metabolic capabilities, including genes for amino acid utilisation, arsenate reduction, and complex carbon degradation—most notably, hexosaminidase for chitin breakdown. Additionally, Verrucomicrobiota_9 encodes FeFe‐ and NiFe‐hydrogenases and genes involved in methane oxidation. The absence of genes for oxygen metabolism in both MAGs further supports their adaptation to anaerobic conditions, highlighting their specialised roles in sustaining the anoxic microbial network. Future work will address the metabolic potential and activity of these MAGs.

### 3.3 Activity Measurements Provide Additional Context for the Identification of Keystone Taxa

We posited that the presence of microbial taxa alone does not necessarily indicate ecological activity. While metagenomic data reveal the genomic potential of taxa, identifying which organisms are present and what they are capable of, it does not capture real‐time functional engagement with the environment. To designate a MAG as a keystone taxon, we proposed that it must not only be present but also transcriptionally active under current environmental conditions. To this end, we incorporated gene expression data from metatranscriptomes, which reflect active functional contributions of microbes. Our robustness analysis showed that Bacteroidota_6 (Ga0485171_metabat1.063) consistently maintained high connectivity in both metagenomic and metatranscriptomic networks across varied community configurations, reinforcing its role as an active and influential keystone taxon. In contrast, Verrucomicrobiota_9 (Ga0485167_metabat2_ours.023) showed limited connectivity in both datasets, indicating a less central role.

These methodologies come with their own set of challenges (e.g., high dimensionality, data sparsity, the compositional nature of microbiome data, and the need for careful normalisation and filtering strategies). Metagenomics, which involves sequencing bulk DNA from the environment, portrays the ‘genomic potential’ of ecosystems. Metagenomic binning reconstructs microbial genomes, linking genomic function to taxonomy (Tran et al. 2023). Most prior work on keystone species identification has focused on amplicon (16S rRNA sequencing) or metagenomic analyses, whereas here we focus on assessing transcriptomics networks to identify keystone species. By integrating these analyses with experimental or quantitative data, we can significantly enhance the resolution of observations and provide deeper insights into community interactions. We applied network analysis methods not only to identify species–environment interactions in a complex, dynamic system, but also to validate ‘hypothetical keystone species’ using mock community simulations and targeting transcriptionally active taxa. Network tools such as SPIEC‐EASI (Kurtz et al. 2015) and CARlasso (Shen and Solis‐Lemus 2020, 2021) rely heavily on statistical correlations, which do not imply causation. Their predicted interactions often require further validation through experimental manipulations or longitudinal studies (Zhou and Ning 2017; Martiny et al. 2006). While the long‐term time series dataset of Lake Mendota lacks transcriptomics data, our present study covering one summer of paired metagenomic–metatranscriptomic data can vouch for the importance of incorporating transcriptomics in future studies. The integration of activity measurements—particularly those derived from metatranscriptomic data—enhances our understanding of microbial community dynamics and keystone taxa identification. This approach can confirm the presence of taxa within a community but importantly, demonstrates their active roles and interactions beyond what metagenomics can inform (Terrón‐Camero et al. 2022). In our study, taxa such as members of Bacteroidota exhibited significant ecological functions that are sometimes only detectable through gene expression profiling, underscoring their roles as keystone species in different network configurations. To place these findings in context with prior work, we compared our results with previously reported freshwater keystone taxa, such as members of Microbacteriaceae, Rhodobacteraceae, and Sphingomonadaceae in the water column and microalgae and protostomes in lake sediments (Ren et al. 2021; Li et al. 2023). We found no direct overlap; none of these previously identified taxa emerged as highly connected nodes in our activity‐informed networks. Instead, our analyses highlighted different lineages, such as Bacteroidota_6, as keystone taxa when considering both abundance and transcriptional activity.

In microbial ecology and keystone species studies, it has often been found that low‐abundance taxa can actually serve as keystone species, since they shape the ecosystems. Metatranscriptomics and environmental sampling that covers distinct ecological niches (oxygen, temperature, etc.) can further help identify these keystone species. Furthermore, our findings align with the emerging consensus in the field that the abundance of a microbe, as suggested by metagenomic data, does not always correlate with its functional impact. As supported by Fan et al. (2012), this discrepancy has been observed in complex microbial communities associated with sponges, where functionally equivalent symbionts were found to be delivered by diverse and phylogenetically distinct taxa. Their study demonstrates that while metagenomic data can reveal the presence and relative abundance of microbial taxa, these structural indicators may not necessarily reflect the microbes' actual ecological or functional roles within their environment. Such distinctions are critical, as discussed in recent influential studies (Banerjee et al. 2018a, 2018b), which advocate a dual perspective on microbial significance in ecosystems. An additional component that can be integrated with microbial metagenomics and metatranscriptomics is viromics (Bikel et al. 2015), which may help unravel the drivers of community dynamics and support a more comprehensive understanding of microbiomes.

## 4 Materials and Methods

### 4.1 Data Description and Data Analysis

#### 4.1.1 Data Collection and Sampling

The data used in this study were previously obtained as part of a distinct, prior study (Tran et al. 2023). Lake Mendota is an eutrophic lake located in Madison, WI, USA. The lake sits at 259 m elevation, covers 3961.2 ha, has a mean depth of 12.8 m, and a maximum depth of 25.3 m. The lake sits low in the landscape, and has a high level of shoreline development. The lake is one of the core study sites of the North Temperate Lakes—Long‐Term Ecological Research (https://lter.limnology.wisc.edu/core‐study‐lakes/) In summary, to assess the impact of anoxia on phage‐host relationships over time, Lake Mendota was previously sampled weekly (once a week) from June to October 2020. During that period, samples were collected at 0, 5, 15, 20, and 23.5 (maximum depth) for DNA, viral DNA and RNA each time, and a YSI profile was collected to capture distinct depth environmental conditions (see Figure 1 and for additional details on the data, see figure 1 in Tran et al. 2023). All the samples were either frozen for DNA and RNA archival purposes, or in the case of a subset of them (those in this study) were extracted for metagenomic and metatranscriptomic sequencing. To choose which samples were to be sequenced, we first plotted the environmental profile, to determine how the oxycline shifted over time. We selected samples throughout the season above, at and below the oxycline to capture changing oxygen and environmental conditions. Additionally, we selected samples before and after fall mixing, which homogenises the water column. Detailed bioinformatics protocols for processing the metagenomes and metatranscriptomes are described in (Tran et al. 2023). In short, reads were quality‐checked, assembled using SPAdes (Bankevich et al. 2012), binned using MetaBat1 (Kang et al. 2015), Metabat2 (Kang et al. 2019), Maxbin2 (Wu et al. 2016), refined using DASTool (Sieber et al. 2018), dereplicated using the bins were dereplicated with the following settings: at 90% for primary clusters, 95% for secondary clusters, and 10% minimum coverage overlap (Olm et al. 2017), quality‐checked using CheckM (Parks et al. 2015) and taxonomically assigned using Genome Taxonomy Database Toolkit (GTDB‐Tk) (Chaumeil et al. 2020). Only MAGs corresponding to MIMAG standards (medium and high‐quality) were used for downstream analyses. The MAG abundance and expression were quantified using CoverM (Aroney et al. 2025) by mapping metagenomic and metatranscriptomic reads back to the MAG, respectively, generating two matrices: a MAG abundance matrix, and a MAG expression matrix. These two matrices are the main input for this current manuscript for the downstream network analyses. Descriptions of environmental variables for all sequenced samples (used in SPEAC‐EASI and CARlasso analyses) are provided in Table S3. A summary of key environmental conditions across all samples, including average, minimum, and maximum values, is presented in Table S5. Taxonomic assignments were obtained with GTDB‐tk; genus names are reported when available. When a genus‐level assignment was not supported, we report the lowest confident rank returned by GTDB‐tk (e.g., Family), labelled as Family (fam) or ‘Unclassified’ in figures/tables.

#### 4.1.2 PERMANOVA Data Analysis

To analyse microbial community composition and assess spatial and temporal variations, we applied Principal Coordinates Analysis (PCoA) and Permutational Multivariate Analysis of Variance (PERMANOVA).

PCoA Analysis is an ordination technique that transforms a distance matrix into a lower‐dimensional representation while preserving dissimilarities between samples. We constructed a Bray–Curtis dissimilarity matrix, which quantifies differences in microbial community composition based on relative abundance. Since Bray–Curtis is sensitive to highly abundant taxa, we first applied Hellinger transformation to the raw abundance data to reduce the influence of dominant species and improve comparability across samples. The first two principal coordinates (PCoA1 and PCoA2) were used to generate a 2D ordination plot, where samples closer together have more similar microbial compositions, while those farther apart are more dissimilar. To statistically assess the influence of depth, seasonal variation and oxygen level on microbial community composition, we conducted PERMANOVA using Bray–Curtis dissimilarity. This test evaluates whether microbial communities significantly differ across sample groups by partitioning variance among categorical predictors. The model included: 1‐depth (four levels: 5, 10, 15, 23.5 m), 2‐month (four levels: July, August, September, October), and 3‐oxygen (oxic, anoxic, oxycline). Before computing Bray–Curtis distances, we applied Hellinger transformation to normalise abundance data and mitigate the impact of dominant taxa. The model was run with 999 permutations to assess significance. Pairwise PERMANOVA comparisons revealed significant microbial composition differences across specific depths, months, and oxygen levels supporting the presence of distinct microbial community structures over environmental conditions.

#### 4.1.3 Identification of the Top 10 Most Abundant MAGs Across Environmental Conditions

To identify the top 10 most abundant metagenome‐assembled genomes (MAGs) for each environmental condition, raw MAG abundance counts were normalised using TSS to obtain relative abundances, allowing direct comparison across samples with different sequencing depths. Samples were then categorised separately by depth (5, 10, 15, 23.5 m), month (July, August, September, October), and oxygen condition (oxic, anoxic, oxycline), resulting in 11 distinct environmental conditions. For each condition, we calculated the average relative abundance of each MAG across all samples assigned to that condition, providing a single representative value. MAGs were then ranked in descending order based on these averages, and the 10 highest‐ranking MAGs were recorded as the ‘top 10’ for that condition. The top 10 sets from all conditions were retained, and we also counted the number of conditions (out of 11) in which each MAG appeared, allowing us to identify both consistently abundant taxa across multiple conditions and those characteristic of specific depths, months, or oxygen levels.

### 4.2 Microbial Network Construction and Environmental Association Analysis

**FIGURE 8:**
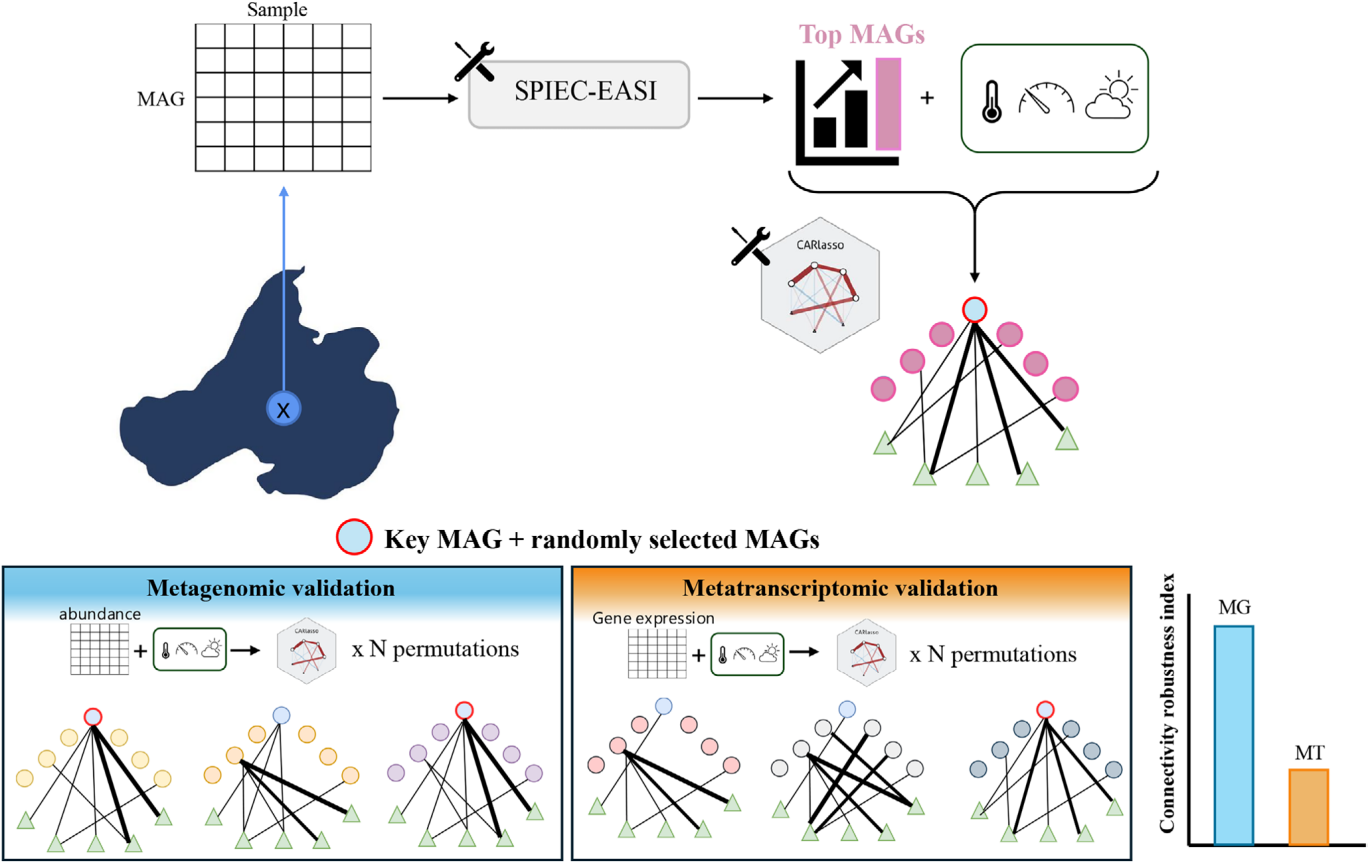
Graphical abstract illustrating the workflow and key findings of this study. We first applied SPIEC‐EASI to metagenomic abundance data to construct microbial co‐occurrence networks and selected the top 15 MAGs based on degree centrality. We then used CARlasso to detect relationships between the top MAGs and environmental conditions. From this set, we selected one key MAG that exhibited strong environmental connectivity for further analysis. To assess the robustness of this key MAG, we performed a permutation‐based analysis by combining it with 14 randomly chosen MAGs and reconstructing networks across 100 permutations. The stability of the key MAG's role was quantified using the ‘connectivity robustness index’, which reflects how consistently it maintained high‐connectivity interactions across different community configurations. Notably, this key MAG showed high centrality in the metagenomic network but not in the metatranscriptomic network, highlighting that abundance‐based significance does not always align with transcriptional activity.

To investigate microbial interactions and their relationships with environmental conditions, we implemented a two‐step approach: (1) we first used SPIEC‐EASI (Kurtz et al. 2015) to identify key MAGs based on their co‐occurrence patterns, and (2) we then applied CARlasso (Shen and Solis‐Lemus 2020, 2021) to examine how these key taxa are influenced by environmental conditions. Figure 8 shows the graphical abstract for this investigation.

#### 4.2.1 Identifying Key Microbial Taxa Using SPIEC ‐ EASI

To identify important microbial taxa, we applied the SPIEC‐EASI algorithm using the graphical LASSO (glasso) method. SPIEC‐EASI was applied directly to the count matrix of MAG abundances, following the usage demonstrated in the official tutorial (e.g., American Gut analysis). The algorithm internally performs normalisation and centered log‐ratio (CLR) transformation to account for the compositional nature of microbiome data, eliminating the need for manual transformation (Kurtz et al. 2015). This method infers conditional dependencies among MAGs by constructing a precision matrix (the inverse covariance matrix), which highlights direct relationships between variables while controlling for others. A non‐zero value in this matrix indicates a direct microbial interaction, independent of other taxa. Using the precision matrix, we built a microbial co‐occurrence network that captures the interdependencies within the microbial community. Feature selection was performed by calculating node connectivity (degree centrality) in the network, which represents the number of direct connections a given MAG has with others. We selected the top 15 MAGs based on their degree values, considering them as the most influential MAGs in terms of network structure and ecological impact (Aghdam et al. 2024). These key MAGs were later used for downstream analyses. While SPIEC‐EASI effectively captures microbial co‐occurrence patterns, it does not incorporate environmental covariates, limiting its ability to assess environmental drivers of microbial variation. To address this limitation, we used our package, CARlasso (Shen and Solis‐Lemus 2020, 2021), to establish direct associations between microbial taxa and environmental conditions.

#### 4.2.2 Linking Microbial Taxa With Environmental Conditions Using CARlasso

To explore the direct associations between microbial taxa and environmental conditions, we employed the CARlasso method (Shen and Solis‐Lemus 2020, 2021), implemented in R. CARlasso operates within a Bayesian framework to infer Gaussian chain graphs, offering a powerful and flexible approach for ecological network analysis. The Gaussian chain graph constructed by CARlasso incorporates (1) correlated responses which represent microbial abundances (in our case, the 15 key MAGs identified via SPIEC‐EASI), and (2) predictors which correspond to environmental or experimental variables that may influence the microbial responses. The model infers conditional dependencies among taxa and predictors, capturing interactions within the microbial community and associations with environmental predictors. For both the metagenomic and metatranscriptomic datasets, we used the same 15 MAGs as response variables generated by running SPIEC‐EASI. In the metagenomic dataset, the response matrix consisted of the abundances of the 15 MAGs across samples. In the metatranscriptomic dataset, the response matrix consisted of the corresponding transcript counts mapped to the same 15 MAGs. We used the same set of predictors for metagenomic and metatranscriptomic datasets, including variables such as dissolved oxygen, temperature, chlorophyll concentration, pH, and nutrient levels (e.g., nitrate, phosphate) (Table S3). This setup enables the model to disentangle complex ecological relationships by simultaneously modelling microbial co‐occurrence patterns and their responses to environmental gradients. To ensure compatibility with the model and comparability across variables, we applied the following normalisation strategies:

- *Microbial responses (MAG abundances)* were normalised internally using the log=TRUE parameter within the CARlasso package, which applies a log‐transformation to account for skewed abundance distributions and compositional effects.
- *Environmental predictors* were normalised using *Min–Max scaling*, transforming each feature to the $\left[0,1\right]$ range using the formula: $X_{\mathrm{Min}‐\mathrm{Max}}=\frac{X-X_{\min}}{X_{\max}-X_{\min}},$ where X is the original value, and $X_{\min}$ and $X_{\max}$ are the minimum and maximum values of the variable. This prevents variables with large numeric ranges from dominating the model and improves interpretability of the estimated associations.

### 4.3 Permutation‐Based Evaluation of Keystone Taxa

To evaluate the robustness of the identified keystone taxa and their centrality within the microbial network, we conducted a permutation‐based analysis. This approach was designed to simulate shifts in microbial community composition that might occur in response to environmental changes, providing insight into the stability of the central taxa under such variations. In our permutation analysis, we first selected the top 15 MAGs based on the highest degree of centrality in the metagenomic dataset by SPIEC‐EASI. We then constructed the CARlasso networks based on these 15 selected MAGs and all environmental conditions in two datasets—metagenomic abundance for the DNA‐based network and metatranscriptomic expression for the RNA‐based (Figure S7). Two representative MAGs are selected for further evaluation based on their distinct connectivity profiles: Ga0485171_metabat1.063 (Bacteroidota_6), which exhibited high centrality in the metagenomic network inferred using CARlasso (Figure S7a), and Ga0485167_metabat2_ours.023 (Verrucomicrobiota_9), which showed greater connectivity in the metatranscriptomic network inferred using CARlasso (Figure S7b). For each selected MAG, we constructed 100 microbial networks using CARlasso, where the selected taxon was retained and combined with 14 randomly selected MAGs. This approach simulates alternative community configurations while enabling repeated evaluation of the selected MAG's role across diverse contexts. To quantify the robustness of a selected MAG's connectivity, we extracted all edge weights from each permuted network and determined the 90th percentile of the edge weight distribution. Any edge involving the selected MAG that exceeded this threshold was considered a high‐connectivity interaction. We then calculated the percentage of permutations in which the selected MAG was involved in high‐connectivity edges. This value served as a metric for the taxon's network centrality across randomized communities; we refer to it as the ‘connectivity robustness index’, which reflects the frequency (ranging from 0% to 100%) with which a taxon participates in high‐connectivity interactions across alternative community configurations. Higher values indicate greater consistency in the taxon's central role across varying microbial networks, suggesting that the MAG maintains high connectivity and ecological importance regardless of which other taxa are present in the community.

## Author Contributions

**Qiyao Yang:** writing – original draft, methodology, software, formal analysis, validation, visualization, investigation. **Rosa Aghdam:** investigation, software, formal analysis, methodology, validation, writing – review and editing, conceptualization. **Patricia Q. Tran:** writing – review and editing, data curation, validation, investigation, conceptualization. **Karthik Anantharaman:** writing – review and editing, supervision, investigation, data curation, conceptualization, project administration, resources. **Claudia Solís‐Lemus:** writing – review and editing, funding acquisition, supervision, investigation, methodology, validation, conceptualization, project administration, resources.

## Funding

This work was supported by a Community Science Program New Investigator award (506328), the Natural Science and Engineering Research Council of Canada (NSERC), the National Science Foundation (DBI‐2047598, DEB‐2144367), the USDA National Institute of Food and Agriculture (Hatch 1025641), the University of Wisconsin‐Madison, the Joint Genome Institute, and the Office of Science.

## Conflicts of Interest

The authors declare no conflicts of interest.

## Supporting information


**Data S1:** Supporting Information.

## Data Availability

The data that support the findings of this study are openly available in Zenodo at https://zenodo.org/records/14046929.
