# Supplementary material for: Activity‐Informed Network Analysis Reveals Keystone Microbes Shaping Freshwater Ecosystem Function
Source: Environ Microbiol Rep. 2026 Mar 19;18(2):e70245. doi: 10.1111/1758-2229.70245 (PMC13053139; doi:10.1111/1758-2229.70245)
Supplement: Supplementary file 1 — Data S1: Supporting Information. [file EMI4-18-e70245-s001.pdf]

SUPPLEMENTARY MATERIAL: Activity-Informed Network Analysis Reveals Keystone Microbes Shaping Freshwater Ecosystem Function

Qiyao Yang[1,2,3,+], **Rosa Aghdam**[1,+], **Patricia Q. Tran**[4], **Karthik Anantharaman**[4,*], **Claudia Solís- Lemus**[1,5,*]

[1]Wisconsin Institute for Discovery, University of Wisconsin-Madison, Madison, WI
[2]Department of Computer Science, University of Wisconsin-Madison, Madison, WI
[3]Department of Statistics, University of Wisconsin-Madison, Madison, WI
[4]Department of Bacteriology, University of Wisconsin-Madison, Madison, WI
[5]Department of Plant Pathology, University of Wisconsin-Madison, Madison, WI
[*]karthik@bact.wisc.edu,solislemus@wisc.edu
[+]These authors contributed equally to this work.

September 24, 2025

List of Tables

1 Pairwise correlation coefficients (Pearson and Spearman) of microbial community composition, showing sample pairs with strong positive correlations ($r > 0.8$ for both methods) 6
2 Description of the node names in the `CARlasso` network diagram, showing the top 15 nodes with the highest degree centrality, selected using the `SPIEC-EASI` algorithm 6
3 Descriptions of environmental parameters used in the study. 7
4 Description of node identities in the `CARlasso` network diagram used for the permutation analysis involving `p_Bacteroidota_6`. 7
5 Summary statistics (average, minimum, and maximum) of environmental variables 8

List of Figures

1 Boxplot for distribution of the abundance of genome per sample before normalization. 2
2 Boxplot for distribution of the relative abundance of MAGs per sample after normalization. . 2
3 Distribution of relative abundance values for MAGs across individual samples. 3
4 Mean relative abundance of metagenome-assembled genomes (MAGs) across sampling groups. 3
5 Pearson correlation heatmap of microbial communities across 16 lake samples. 4
6 Spearman correlation heatmap of microbial communities across 16 lake samples 5
7 Network diagrams of microbial-environment interactions 8

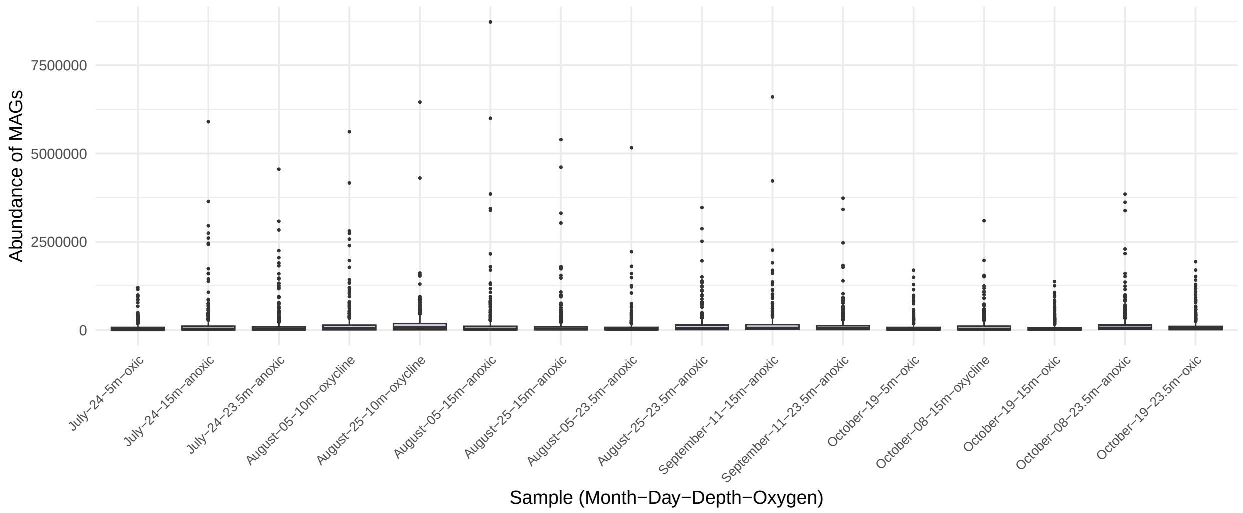

Figure 1: Boxplot showing the distribution of MAGs abundance per sample before normalization. Each box represents the abundance values of all MAGs within a given sample. The x-axis labels follow the format Month-Day-Depth-Oxygen, indicating the sampling date, depth (in meters), and oxygen condition. For example, the label July-24-15m-Oxic refers to a sample collected on July 24 at a depth of 15 meters under oxic conditions.

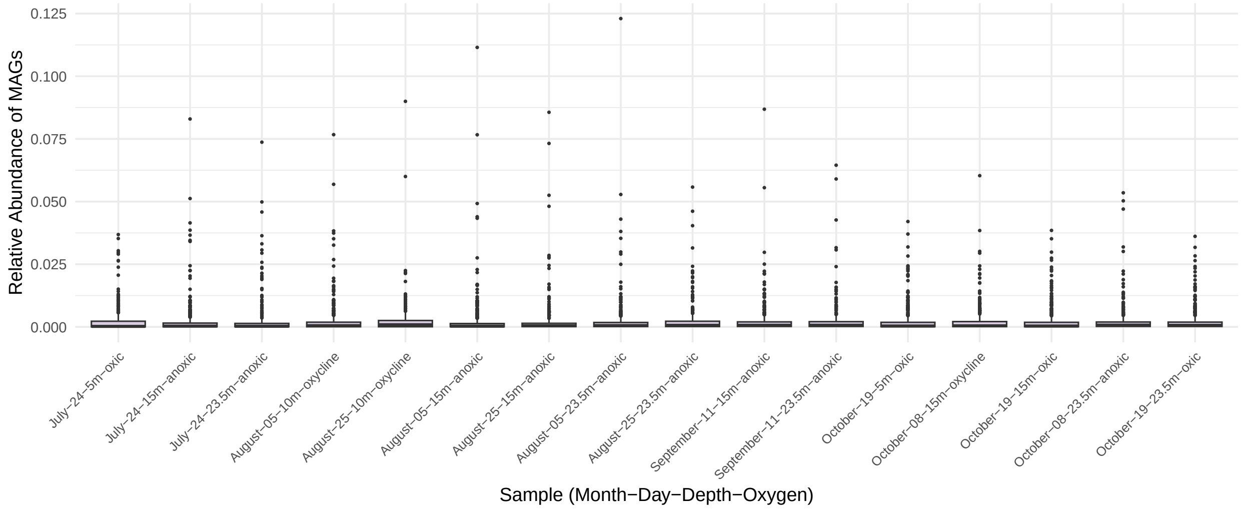

Figure 2: Boxplot showing the distribution of relative abundance of MAGs per sample after normalization. Each box represents the relative abundance values of all MAGs within a given sample. The x-axis labels follow the format Month-Day-Depth-Oxygen, indicating the sampling date, depth (in meters), and oxygen condition. For example, the label July-24-15m-Oxic refers to a sample collected on July 24 at a depth of 15 meters under oxic conditions.

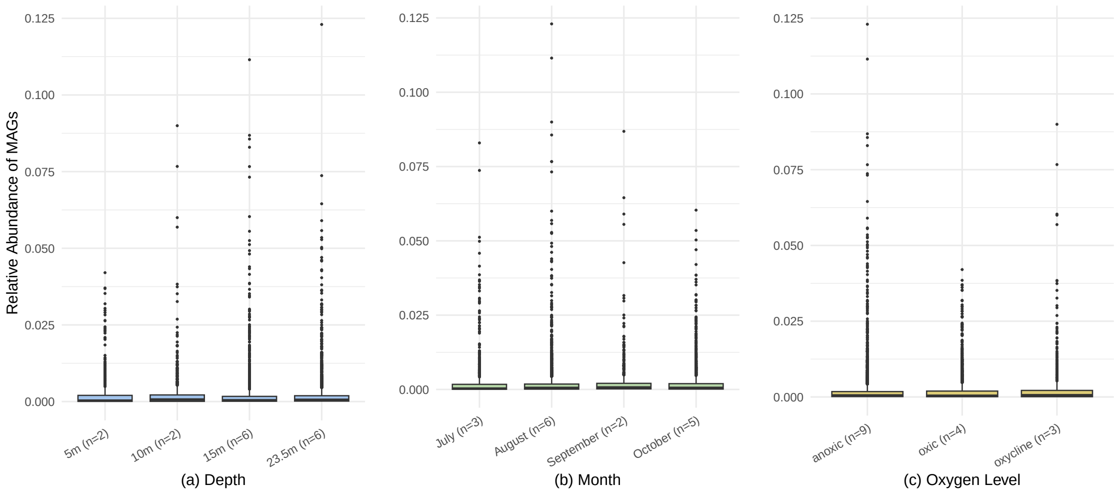

Figure 3: Distribution of relative abundance values for MAGs across individual samples, grouped by (a) depth, (b) month, and (c) oxygen condition. Relative abundances were calculated using total sum scaling normalization per sample. Each point in the boxplot represents a single MAG's abundance in one sample, totaling 431 values per sample. Boxplots summarize the spread of MAG abundances within each group, and sample sizes are indicated on the x-axis.

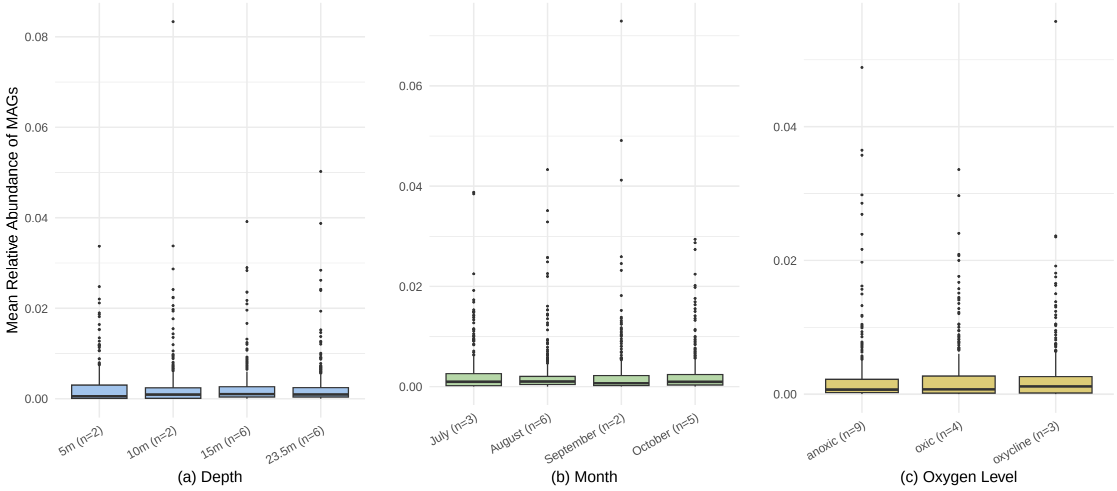

Figure 4: Mean relative abundance of metagenome-assembled genomes (MAGs) across sampling groups. Relative abundances were calculated using total sum scaling normalization per sample. For each group defined by (a) depth, (b) month, and (c) oxygen condition, we calculated the mean relative abundance of each MAG by averaging its normalized abundance across all samples in the group. Each boxplot represents the distribution of these averaged values for 431 MAGs. Sample sizes for each group are shown on the x-axis.

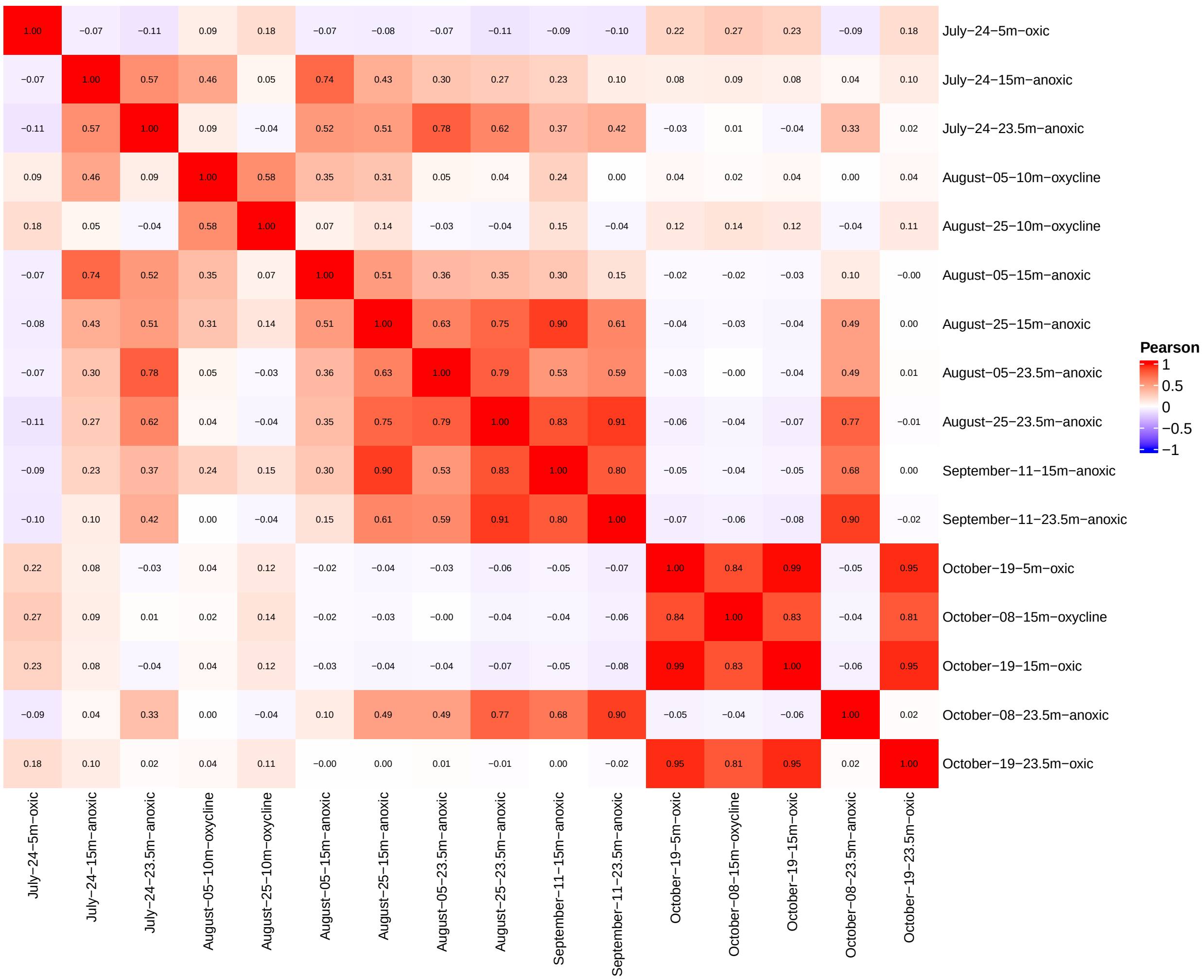

Figure 5: Pearson correlation heatmap of microbial communities across 16 lake samples. Pairwise Pearson correlation coefficients were calculated based on the relative abundance of 431 MAGs per sample. The color scale represents correlation strength and direction: red indicates strong positive correlation, blue indicates negative correlation, and white indicates weak or no correlation. Sample labels follow the "Month–Day–Depth–Oxygen" format and are ordered by sampling date and depth.

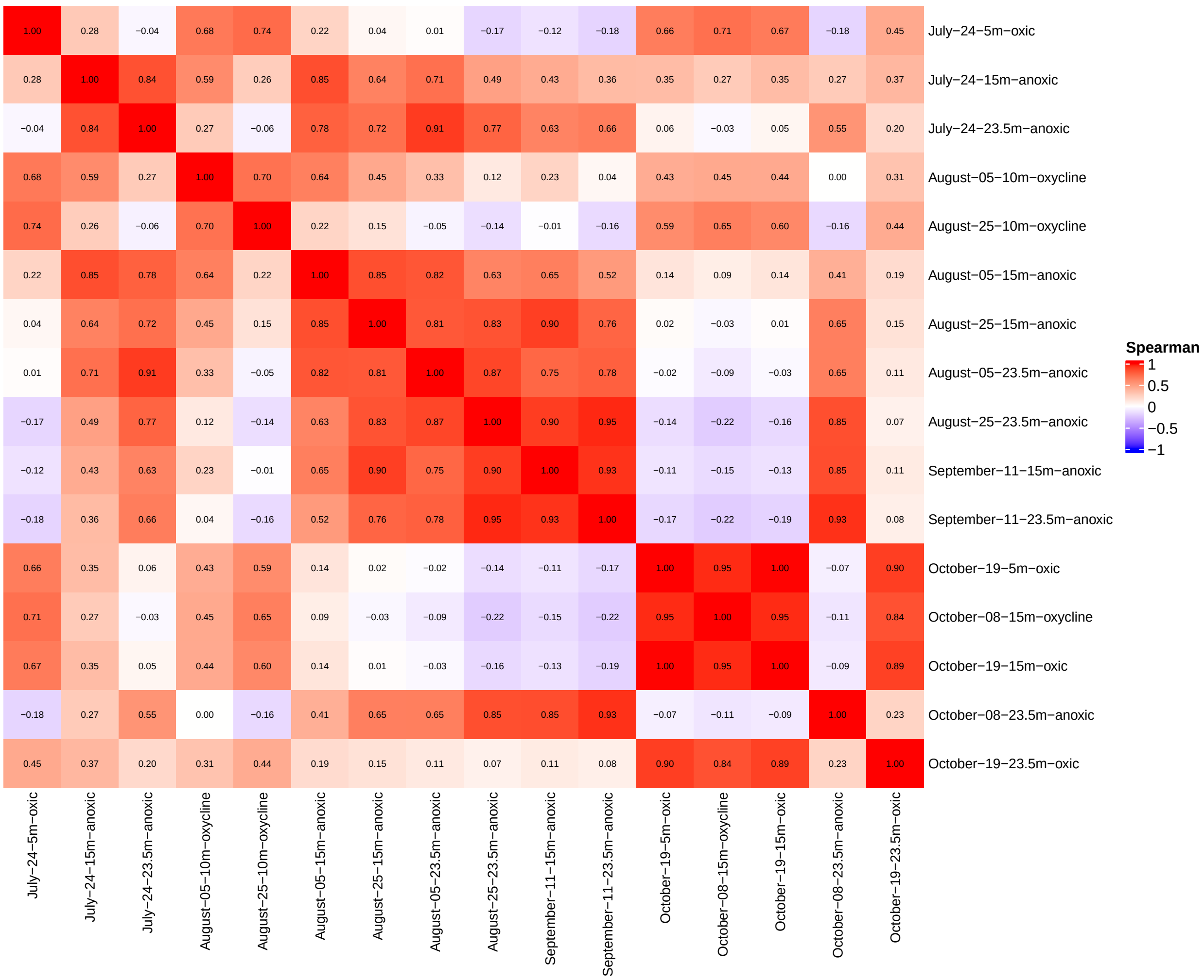

Figure 6: Spearman correlation heatmap of microbial communities across 16 lake samples. Pairwise Spearman correlation coefficients were calculated based on the relative abundance of 431 MAGs per sample. The color scale represents correlation strength and direction: red indicates strong positive correlation, blue indicates negative correlation, and white indicates weak or no correlation. Sample labels follow the "Month–Day–Depth–Oxygen" format and are ordered by sampling date and depth.

Table 1: Pairwise correlation coefficients (Pearson and Spearman) of microbial community composition, showing sample pairs with strong positive correlations ($r > 0.8$ for both methods)

#	**Sample 1**	**Sample 2**	**Pearson**	**Spearman**
1	August-25-15m-anoxic	September-11-15m-anoxic	0.902	0.896
2	August-25-23.5m-anoxic	September-11-15m-anoxic	0.827	0.900
3	August-25-23.5m-anoxic	September-11-23.5m-anoxic	0.910	0.952
4	October-08-15m-oxycline	October-19-15m-oxic	0.829	0.947
5	October-08-15m-oxycline	October-19-23.5m-oxic	0.811	0.836
6	October-08-15m-oxycline	October-19-5m-oxic	0.843	0.947
7	October-08-23.5m-anoxic	September-11-23.5m-anoxic	0.897	0.933
8	October-19-15m-oxic	October-19-23.5m-oxic	0.948	0.890
9	October-19-15m-oxic	October-19-5m-oxic	0.993	0.998
10	October-19-23.5m-oxic	October-19-5m-oxic	0.946	0.898
11	September-11-15m-anoxic	September-11-23.5m-anoxic	0.803	0.928

Table 2: Description of the node names in the `CARlasso` network diagram, showing the top 15 nodes with the highest degree centrality, selected using the `SPIEC-EASI` algorithm [24]. This table serves as a reference for the response nodes in Figure 2. The "MAG identifier" column lists the MAG names, and the Phylum column corresponds to the node labels used in the network for easy cross-reference. The remaining columns provide taxonomic and genomic details for each MAG. The "Size" column represents genome size in megabase pairs (Mbp), and the "Com(%)" column indicates completeness percentage.

**MAG identifier**	**Phylum**	**Domain**	**Class**	**Order**	**Family**	**Genus**	**Size**	**Com(%)**
Ga0485158_metabat2_ours.098	Krumholzibacteriota_1	Bacteria	Krumholzibacteria	LZORAL124-64-63	LZORAL124-64-63	CAINDZ01	3.75	88.03
Ga0485159_metabat2_ours.079	Bacteroidota_2	Bacteria	Bacteroidia	Chitinophagales	Chitinophagaceae	Sediminibacterium	1.24	64.08
Ga0485171_metabat2_ours.127_sub	Bacteroidota_3	Bacteria	Bacteroidia	Chitinophagales	Chitinophagaceae	Sediminibacterium	1.33	57.55
Ga0485171_metabat2_ours.004	Proteobacteria_4	Bacteria	Gammaproteobacteria	Burkholderiales	Burkholderiaceae	Polynucleobacter	1.28	68.69
Ga0485170_maxbin.090	Planctomycetota_5	Bacteria	Phycisphaerae	Phycisphaerales	SM1A02	UBA966	1.79	70.09
Ga0485171_metabat1.063	Bacteroidota_6	Bacteria	Bacteroidia	Chitinophagales	LD1		2.15	87.68
Ga0485157_metabat2_ours.019	Chloroflexota_7	Bacteria	Ellin6529	CSP1-4	UBA10416	UBA10416	0.95	62.35
Ga0485166_metabat2_ours.038	Bacteroidota_8	Bacteria	Bacteroidia	Bacteroidales	FEN-979	CAIVAT01	4.17	85.62
Ga0485167_metabat2_ours.023	Verrucomicrobiota_9	Bacteria	Kiritimatiellae	CAIKKV01	CAITUY01	CAITUY01	2.50	58.7
Ga0485170_maxbin.059_sub	Actinobacteriota_10	Bacteria	Acidimicrobiia	Acidimicrobiales	Ilumatobacteraceae	UBA2093	1.63	53.45
Ga0485171_maxbin.130_sub	Planctomycetota_11	Bacteria	Phycisphaerae	Phycisphaerales	SM1A02	UBA966	2.45	77.22
Ga0485160_metabat2_ours.158_sub	Chloroflexota_12	Bacteria	Ellin6529	CSP1-4	UBA10416	UBA10416	0.70	52.33
Ga0485161_maxbin.110	Proteobacteria_13	Bacteria	Gammaproteobacteria	Burkholderiales	Rhodocyclaceae		3.71	84.5
Ga0485161_metabat1.096	Firmicutes_A_14	Bacteria	Clostridia	Saccharofermentanales	UBA5734	CAILLO01	1.91	79.18
Ga0485161_metabat2_ours.167_sub	Verrucomicrobiota_15	Bacteria	Kiritimatiellae	RFP12	UBA1067	CAIZQW01	6.05	84.96

Table 3: Descriptions of environmental parameters used in the study.

Parameter	Units	Description
depth	m	Represents the depth in meters at which each sample was taken. This is critical for studying the vertical stratification of microbial communities and their response to depth-dependent variables.
wtemp	°C	Water temperature at the time of sampling, measured in Celsius. Temperature is a primary ecological factor that influences metabolic rates and the distribution of microbial species.
do_raw	mg/L	The raw measurement of dissolved oxygen in milligrams per liter, providing a direct quantification of oxygen in the sampled environment.
sp_cond	µS/cm	Specific conductivity measured in microsiemens per centimeter, which helps assess the water's ionic strength and its effects on microbial osmotic balance.
pH		The hydrogen ion concentration, indicating the acidity or alkalinity of the water. pH can significantly affect microbial survival and biochemical pathways.
chlor_rfu	RFU	Chlorophyll fluorescence in relative fluorescence units, a proxy for the biomass of phytoplankton, which forms the base of the aquatic food web.
phyco_rfu	RFU	Phycocyanin fluorescence in relative fluorescence units, used to estimate the abundance of cyanobacteria, crucial primary producers in many aquatic systems.
fdom_rfu	RFU	Fluorescence of dissolved organic matter, measured in relative fluorescence units. This parameter reflects the concentration of organic compounds that serve as a substrate for heterotrophic microbes.
turb_fnu	RFU	Turbidity in relative fluorescence units, indicating the clarity of water which affects light penetration and can influence microbial activity.

Table 4: Description of node identities in the `CARlasso` network diagram used for the permutation analysis involving `p_Bacteroidota_6`. This table serves as a reference for the response nodes in Figure 3. The "MAG identifier" column lists the MAG names, and the Phylum column corresponds to the node labels used in the network for easy cross-reference. The remaining columns provide taxonomic and genomic details for each MAG. The "Size" column represents genome size in megabase pairs (Mbp), and the "Com(%)" column indicates completeness percentage.

MAG identifier	Phylum	Domain	Class	Order	Family	Genus	Size	Com(%)
Ga0485159_maxbin.004_sub	Cyanobacteria_1	Bacteria	Cyanobacteriia	PCC-6307	Cyanobiaceae	Cyanobium	2.72	76.04
Ga0485169_maxbin.153	Proteobacteria_2	Bacteria	Gammaproteobacteria	Burkholderiales	Rhodocyclaceae	Azonexus	3.16	82.13
Ga0485163_maxbin.002_sub	Planctomycetota_3	Bacteria	UBA1135	UBA1135	GCA-002686595	SYGM01	4.04	97.85
Ga0485159_metabat2_ours.130_sub	Bacteroidota_4	Bacteria	Bacteroidia	Flavobacteriales	UA16		1.70	65.59
Ga0485164_metabat1.042_sub	Proteobacteria_5	Bacteria	Gammaproteobacteria	Burkholderiales	Burkholderiaceae	Rhodoferax	3.36	84.27
Ga0485171_metabat1.063	Bacteroidota_6	Bacteria	Bacteroidia	Chitinophagales	LD1		2.15	87.68
Ga0485162_metabat2_ours.088	Actinobacteriota_7	Bacteria	Actinomycetia	Nanopelagicales	UBA5976	UBA5976	1.07	66.07
Ga0485161_maxbin.064	Actinobacteriota_8	Bacteria	Actinomycetia	Nanopelagicales	Nanopelagicaceae	Planktophila	1.17	72.7
Ga0485172_metabat2_ours.152	Actinobacteriota_9	Bacteria	Actinomycetia	Nanopelagicales	Nanopelagicaceae	UBA3012	0.998	68.07
Ga0485161_metabat2_jgi.003_sub	Verrucomicrobiota_10	Bacteria	Verrucomicrobia	Verrucomicrobiales	Akkermansiaceae	UBA1315	3.74	91.8
Ga0485168_metabat2_ours.010	Proteobacteria_11	Bacteria	Alphaproteobacteria	Caulobacterales	Hyphomonadaceae	UBA7672	3.29	84.05
Ga0485157_metabat2_ours.068_sub	Bacteroidota_12	Bacteria	Bacteroidia	AKYH767	SXHM01		2.53	64.11
Ga0485169_maxbin.226_sub	Verrucomicrobiota_13	Bacteria	Kiritimatiellae	CAIKKV01	CAITUY01	CAITUY01	3.28	65.2
Ga0485169_metabat2_ours.154	Firmicutes_A_14	Bacteria	Clostridia	Saccharofermentanales	UBA5734		1.98	75.66
Ga0485160_maxbin.164_sub	Bacteroidota_15	Bacteria	Bacteroidia	Bacteroidales	CAIXZF01	CAIXZF01	2.63	66.38

Table 5: Summary statistics (average, minimum, and maximum) of environmental variables

**Variable**	**Average**	**Minimum**	**Maximum**
Depth (m)	16.31	5.00	23.50
Water Temperature (°C)	13.56	10.31	25.05
DO Saturation (%)	19.87	-1.70	109.10
DO Raw (mg/L)	1.93	-0.18	9.00
Specific Conductivity (μS/cm)	575.33	492.70	627.90
pH	7.61	7.17	8.62
Chlorophyll (RFU)	0.40	0.09	0.99
Phycocyanin (RFU)	0.78	0.34	1.70
fDOM (RFU)	11.98	9.70	13.77
Turbidity (FNU)	1.59	0.59	4.99

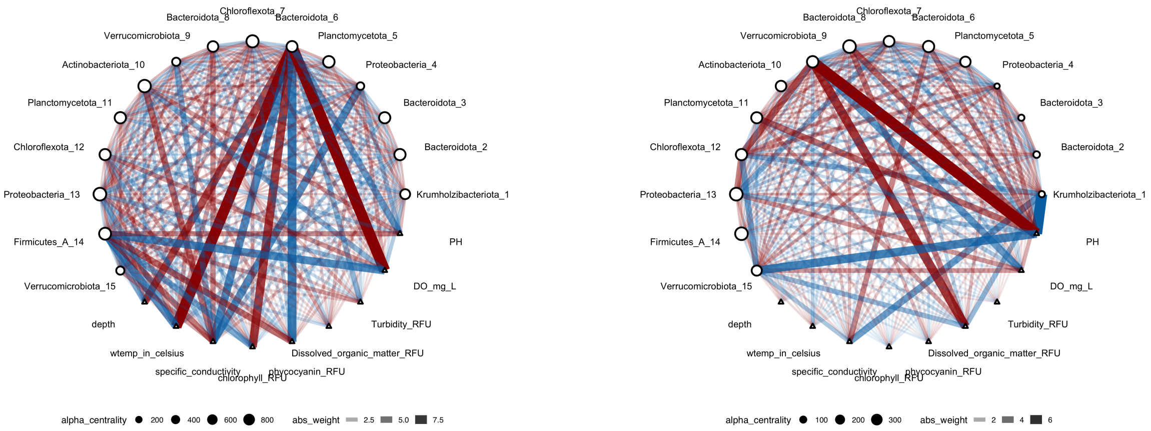

(a) (b)

Figure 7: Network diagrams illustrating the associations between MAGs and environmental variables using the `CARlasso` model [26,27]. In both panels, the top 15 nodes with the highest degree centrality—selected via the `SPIEC-EASI` algorithm [25]—are shown, with node size indicating degree centrality and edge thickness representing interaction strength. Edge colors denote relationship direction (blue: negative; red: positive). Nodes represent MAGs categorized by Phylum and environmental features. (a) Network constructed from metagenomic data, capturing potential ecological roles of MAGs based on their genomic presence. (b) Network constructed from metatranscriptomic data, reflecting active microbial interactions and transcriptional responses to environmental factors.
